# Effect of combined home-based, overground robotic-assisted gait training and usual physiotherapy on clinical functional outcomes in people with chronic stroke: A randomized controlled trial

**DOI:** 10.1177/0269215520984133

**Published:** 2020-12-27

**Authors:** Amy Wright, Keeron Stone, Louis Martinelli, Simon Fryer, Grace Smith, Danielle Lambrick, Lee Stoner, Simon Jobson, James Faulkner

**Affiliations:** 1 School of Sport, Health & Community, University of Winchester, Winchester, UK; 2 School of Sport & Exercise, University of Gloucestershire, Gloucester, UK; 3 Hobbs Rehabilitation, Martyr Worthy, Winchester, UK; 4 Department of Sport and Exercise Sciences, University of Chester, Chester, UK; 5 Faculty of Health Sciences, University of Southampton, Southampton, UK; 6 School of Sport and Exercise, University of North Carolina, Chapel Hill, USA

**Keywords:** Stroke, rehabilitation, robotics, home-based

## Abstract

**Objectives:**

To assess the effect of a home-based over-ground robotic-assisted gait training program using the AlterG Bionic Leg orthosis on clinical functional outcomes in people with chronic stroke.

**Design:**

Randomized controlled trial.

**Setting:**

Home.

**Participants:**

Thirty-four ambulatory chronic stroke patients who recieve usual physiotherapy.

**Intervention:**

Usual physiotherapy plus either (1)10-week over-ground robotic-assisted gait training program (*n* = 16), using the device for ⩾30 minutes per day, or (2) control group (*n* = 18), 30 minutes of physical activity per day.

**Measurements:**

The primary outcome was the Six-Minute Walk Test. Secondary outcomes included: Timed-Up-and-Go, Functional Ambulation Categories, Dynamic Gait Index and Berg Balance Scale. Physical activity and sedentary time were assessed using accelerometry. All measurements were completed at baseline, 10 and 22 weeks after baseline.

**Results:**

Significant increases in walking distance were observed for the Six-Minute Walk Test between baseline and 10 weeks for over-ground robotic-assisted gait training (135 ± 81 m vs 158 ± 93 m, respectively; *P* ⩽ 0.001) but not for control (122 ± 92 m vs 119 ± 84 m, respectively). Findings were similar for Functional Ambulation Categories, Dynamic Gait Index and Berg Balance Scale (all *P* ⩽ 0.01). For over-ground robotic-assisted gait training, there were increases in time spent stepping, number of steps taken, number of sit-to-stand transitions, and reductions in time spent sitting/supine between baseline and 10 weeks (all *P* < 0.05). The differences observed in all of the aforementioned outcome measures were maintained at 22 weeks, 12 weeks after completing the intervention (all *P* > 0.05).

**Conclusion:**

Over-ground robotic-assisted gait training combined with physiotherapy in chronic stroke patients led to significant improvements in clinical functional outcomes and physical activity compared to the control group. Improvements were maintained at 22 weeks.

## Introduction

Robotic devices provide high-intensity, repetitive, task-specific therapy and have been shown to improve gait quality (i.e., stride length, step length), functional outcomes (i.e. walking speed and walking capacity) and motor performance in stroke patients.^1^ Stroke patients with greater functional ability may benefit more from over-ground robotic-assisted gait training opposed to treadmill-based robotics (e.g., Lokomat and LOPES) and end effector devices, which moves the patients in a gait like pattern driven by two movable footplates (e.g., G-EO).

Over-ground robotic-assisted devices allow the patient to walk in a real-world environment,^2^ encourages trunk and balance control,^3^ and allows for substantial kinematic variability while still ensuring successful task execution. A small number of training case series^4–6^ and a single randomized controlled trial^7^ have found modest functional benefits^7^ and improvements in gait speed, endurance, and balance after completing an over-ground robotic-assisted gait training intervention, lasting ⩽ six weeks.^4–6^ However, interventions which last greater than eight weeks may accelerate walking gains and improve functional capacity.^8^ It is plausible that longer programs may elicit greater functional improvements in stroke patients.

The primary use of robotic devices is within a clinical setting, as many available systems are not yet developed for a home-based environment and/or require a trained therapist to operate them. Robotic devices are expensive and research is needed to establish the benefit to cost ratio, and potential risk of harm associated with a device if used within a home-based environment. The “at home” potential, however, of an over-ground robotic-assisted gait training device could potentially improve the efficiency of therapy treatments enabling physiotherapists to implement rehabilitation without being physically present.^9^ Home-based settings may also be efficacious as patients could use such devices more frequently in a familiar context^10^ contributing to the formation of habits leading to long-term behavior change.^11^ Further research is needed to investigate the feasibility, efficacy and application of over-ground robotic-assisted gait training in a home-based environment.

The purpose of this study was to assess the effect of a 10-week home-based rehabilitation program using a lower limb dynamic over-ground robotic-assisted gait training device, in combination with usual care physiotherapy, in ambulatory stroke patients on clinical functional outcomes. It was hypothesized that regular participation in a 10-week over-ground robotic-assisted gait training program would improve functional outcomes in individuals living with stroke. A secondary hypothesis was that over-ground robotic-assisted gait training would increase physical activity and reduce sedentary behavior.

## Methods

This study was a dual-center, parallel group, randomized controlled clinical trial, reported in accordance with CONSORT (Consolidated Standards of Reporting Trials) guidelines.^12^ The study protocol received institutional ethical approval from the University of Winchester (Approval number BLS/16/16) and was registered with the Clinical Trials.gov Protocol Registration and Results System (NCT03104127). The study was funded by the University of Winchester. AlterG Bionic Leg orthoses were provided freely by AlterG (Bionic Leg orthosis, Fremont, CA, USA) who had no input or influence on the data analysis or manuscript preparation. Recruitment started in April 2017 and ended in July 2019.

Participants with chronic stroke (> three months since stroke diagnosis) were identified, screened for eligibility, which included a health history questionnaire, and recruited from a single neuro-physiotherapy practice (Hobbs Rehabilitation, Winchester, UK). All participants were diagnosed with stroke by a specialist neurologist/stroke consultant from a UK National Health Service (NHS) Trust and had undertaken normal inpatient and outpatient rehabilitation in accordance with recommended guidelines.^13^ Eligible participants were contacted by telephone and invited to attend a baseline assessment at the University. Written informed consent was obtained from all participants prior to the commencement of the study.

Study inclusion criteria were: individuals who were between 3 months and 5 years post-stroke at the time of study enrolment, who were community-dwelling, medically stable, and cognitively capable, able to stand and step with an aid or with assistance (defined as a Functional Ambulation Categories between 2 and 5),^14^ and who were either currently receiving physiotherapy or attending a community-based, stroke support group. Exclusion criteria were: Unresolved deep vein thrombosis, unstable cardiovascular conditions, open wounds, active drug resistant infection, recent fractures of involved limb, peripheral arterial disease, incontinence, severe osteoporosis, and/or non-weight bearing.

Participants completed a baseline assessment and follow-up assessments at 10 and 22 weeks after baseline. On completion of the baseline assessment, participants were randomized to either:

(i) a 10-week home-based over-ground robotic-assisted gait training program, including weekly “usual care” physiotherapy (O-RAGT).
(ii) a 10-week “usual care” physiotherapy only program (CON).

Web-based randomization was prepared by an independent researcher with no clinical involvement in the trial, using covariate adaptive randomization.^15^ In this study, participants were sequentially assigned to over-ground robotic-assisted gait training or control by taking into account the following covariates:

(i) Baseline postural sway (only able to stand with an aid versus able to stand unaided; able to stand ⩽two minutes versus able to stand > two minutes).
(ii) Age (age ⩾ 70 years vs <70 years).

The independent researcher informed the participant of group allocation at the end of the baseline assessment. Although participants and the primary researcher collecting outcome data were aware of the allocated treatment condition, in order to control and minimize investigator bias, data analysts were kept blinded to the allocation using an independent researcher to re-code the original data sets before returning the data to the data analyst. Identical assessments to those implemented at baseline, were administered at 10 and 22 weeks after baseline.

Participants were asked to abstain from any moderate-to-strenuous physical activity 24 hours prior to the baseline assessment. During this assessment, a series of clinical functional outcomes were measured, wherein participants could use walking aids (e.g., canes, orthoses) if necessary. The primary outcome for this study was the Six Minute Walk Test as it provides an overall measure of an individual’s walking ability, indicates physical incapacity, and is sensitive to change as a result of rehabilitation therapy which targets walking performance.^16^ The Six Minute Walk Test was conducted indoors on a flat walkway. Participants were required to walk between two cones 10m apart for a total of six minutes and were instructed to complete as far a distance as possible. At the end of the Six Minute Walk Test, participants also reported their terminal Ratings of Perceived Exertion (RPE).^17^

The secondary outcomes included: the Timed-Up-and-Go,^18^ Dynamic Gait Index,^19^ Berg Balance Scale,^20^ Functional Ambulation Categories,^14^ Modified Rankin Scale^21^ and accelerometry (ActivPAL3™ device, PAL Technologies Ltd., Glasgow, Scotland). The ActivPAL3 is an electronic logger that uses static and dynamic accelerometry data to distinguish between sitting/lying, standing, and stepping. The ActivPAL3 device was wrapped in a protective Tegaderm™ (3M, St Paul, USA) and attached to the anterior aspect of the upper third of the thigh, on the asymptomatic side. Participants wore the ActivPAL3 for seven consecutive days and nights. This process was repeated at the 10- and 22-week assessment. The physical activity data were categorized by the ActivPAL3 as: (1) percentage of time spent sitting or lying, (2) percentage of time spent standing, (3) percentage of time spent stepping, and (4) step counts.

**Figure 1.**
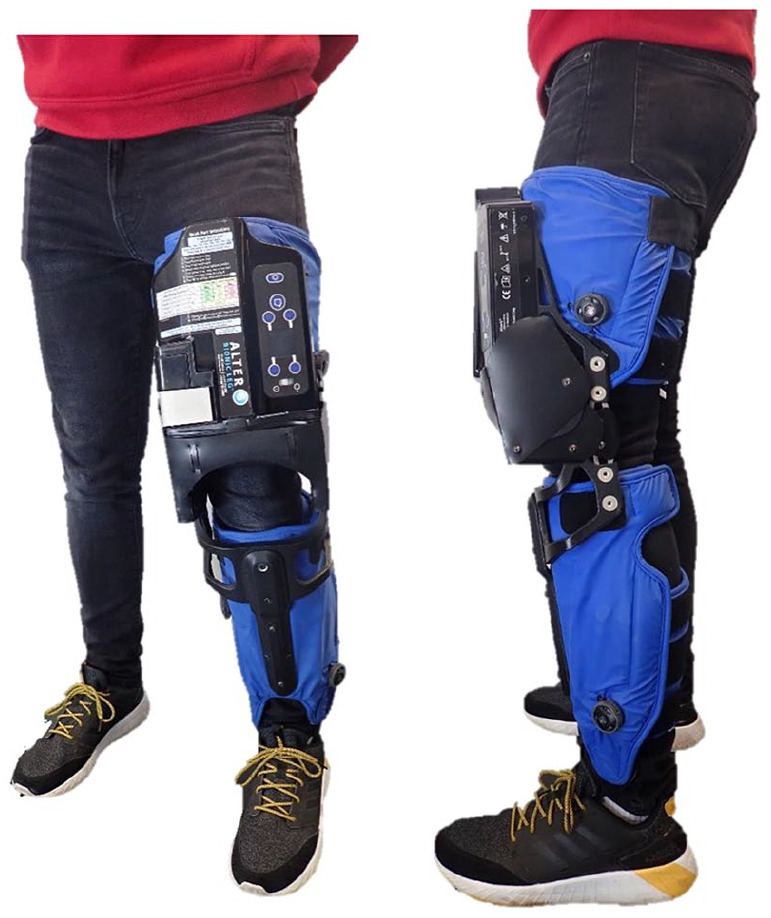
Front and side view of the over-ground robotic-assisted gait training device (Alter G Bionic Leg orthosis, Fremont, CA, USA).

The over-ground robotic-assisted gait training device (Alter-G, Bionic Leg orthosis, Fremont, CA, USA) is an externally-wearable, battery-operated dynamic device that helps patients and therapists during rehabilitation by providing adjustable and progressive functional mobility training (Figure 1). The device consists of an orthosis shell and an actuation unit. The orthosis shell functions as the user interface that transfers the assistive torque to the human body, while the actuation unit assists the movement of the limb which has been shown to be stable, smooth and similar to biological knee motion during sit-to-stand exercises.^6^ The over-ground robotic-assisted gait training device acts to supplement existing muscle strength, provide sensory inputs (i.e. auditory and sensory feedback) and mobility assistance for users with impaired lower-extremity function during rehabilitation (see Supplemental Information), and is fitted and worn in a manner similar to an orthopedic knee brace.

All participants randomized into the over-ground robotic-assisted gait training program completed a familiarization session before taking the device home for the 10-week program period. Participants were advised to wear the device for a minimum of 30 minutes per day, to align with daily physical activity guidelines for older adults, for the purposes of walking and sit-to-stand exercises. The physical activity bouts were not required to be continuous in nature. Participants were advised to exercise at a moderate perception of exertion (RPE 12-13). Although recorded, no daily maximum wear-time was imposed on the participants. Settings for over-ground robotic-assisted gait training device were individualized for each participant, consisting of participant’s weight, assistance, resistance, threshold and knee extension angle settings (see Supplemental Information). Participants’ progress with the over-ground robotic-assisted gait training device was assessed at weeks 2, 4, 6, and 8, and assistance and threshold settings were altered accordingly to elicit progressive overload. Over-ground robotic-assisted gait training participants were provided with a physical activity diary whereby the number of steps, duration of use and activities undertaken were recorded daily. Participants’ compliance in using the robotic device in the over-ground robotic-assisted gait training condition was reported, including the number of days wear, steps and wear-time duration. The average RPE for each day the device was worn was also recorded. During this time, participants also continued their “usual care” physiotherapy (as outlined below).

Participants in both the control group and over-ground robotic-assisted gait training program undertook “usual care” physiotherapy at their local physiotherapy practice. During one-to-one sessions, participants engaged in soft-tissue massage, stretching and muscle strengthening exercises with a therapist, followed by functional movement activities such as sit-to-stand, step-ups, side steps, balance practice, walking, reaching and gripping. Group therapy activities were based on the same principles but with less “hands on” engagement by the therapist. Participants were advised to engage in a minimum of 30 minutes of physical activity each day for the duration of the 10-week program, undertaking similar functional movement patterns as those reported above.

Based on the findings of Ivey et al.,^22^ and when using a two-tailed 5% significance level and a power of 80%, a sample size of 18 per group was calculated to detect a mean difference of 32 m (pooled SD; 45 m) for the Six Minute Walk Test between groups. This calculation incorporated a 20% drop-out rate.

Independent samples *t*-tests were used to compare participant demographics and all clinical functional outcomes at baseline between conditions (over-ground robotic-assisted gait training, control). A series of mixed model 2-factor repeated-measures analysis of variance (ANOVA): Condition (over-ground robotic-assisted gait training, control) × Time (baseline, 10-week, 22-week) were used to assess all clinical functional outcomes and physical activity data. Where statistical differences were observed using ANOVA, post hoc analyses for multiple comparisons were conducted (*t*-tests; Tukey’s honestly significant difference [HSD]). Bonferroni adjustments were used where applicable to reduce the risk of incurring type I error. An intention-to-treat (ITT) analysis was used on all repeated-measures statistical procedures, whereby the last recorded data from a participant’s subsequent assessment was carried forward and used in place of any missing assessments thereafter. Partial eta-squared ( $\eta_{p}^{2}$ ) was used as a measure of effect size, with 0.0099, 0.0588, and 0.1379 representing a small, medium, and large effect.^23^ All calculations were performed using the SPSS 26.0 Software for Windows (SPSS Inc., Chicago, IL, USA).

## Results

**Figure 2.**
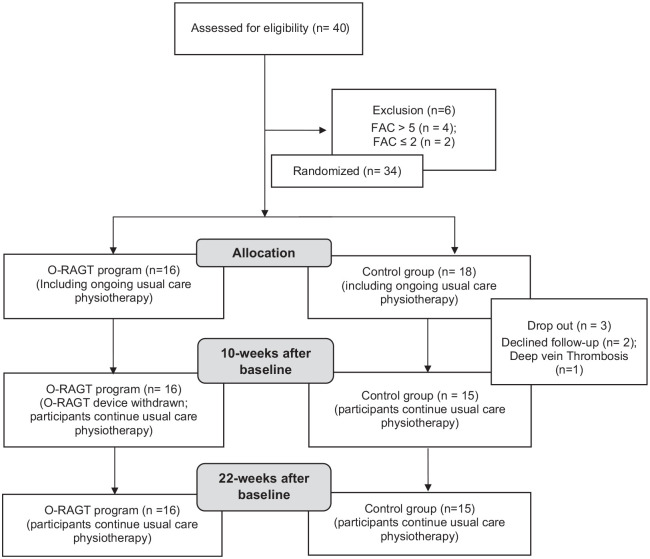
Consort statement. FAC: Functional Ambulation Categories; O-RAGT: Over-ground Robotic-Assisted Gait Training Program using a wearable knee orthosis, Alter G Bionic leg.

Participant recruitment and retention are presented in Figure 2. Thirty-four participants took part in the study (Table 1). The device settings at the start, midpoint and end of the over-ground robotic-assisted gait training program, and participant compliance are presented in Table 2. At the start of the program, participants wore the over-ground robotic-assisted gait training device for six days per week, but by week 10 daily engagement had decreased to five days per week. There was an increase in daily wear time and steps taken, and a decrease in RPE between weeks 1 and 10. Similarly, there were reductions in the “assistance” and increases in the “threshold” settings of the over-ground robotic-assisted gait training device between weeks 1 and 10 (Table 2). There were no adverse events whilst participants wore the over-ground robotic-assisted gait training device. Two participants did not complete the full 10-week program due to difficulties in attending assessments and an unrelated musculoskeletal injury.

**Table 1.** Participant demographics at baseline.

Demographic		O-RAGT		CON		*P* value
		*n*	%	*n*	%	
Gender	Male	14	88	14	78	0.473
	Female	2	12	4	22	
Age (years)		59.6 ± 10.1		65.1 ± 10.1		0.179
Stroke diagnosis	Ischemic	15	94	14	78	0.189
	Hemorrhagic	1	6	4	22	
Hemiparetic side	Left	11	69	10	56	0.445
	Right	5	31	8	44	
Orthotic*	Yes	9	56	10	56	0.969
	No	7	44	8	44	
Walking aid**	Yes	14	88	13	72	0.277
	No	2	12	5	28	
Time since stroke (months)		31 ± 19		32 ± 21		0.877
FAC		3.4 ± 1.0		3.4 ± 1.1		0.970
MRS		3.3 ± 0.6		3.3 ± 0.7		0.874

Age, time since stroke, FAC, and MRS are presented as mean ± SD. All other demographics are presented as total number and percentage.

CON: Control group; FAC: Functional Ambulation Categories; MRS: Modified Rankin Scale; O-RAGT: Over-ground-Robotic Assisted Gait Training.

* Orthotic refers to a soft or hard foot and/or ankle brace.

** Walking aid refers to use of a walking stick, tripod or quadripod.

**Table 2.** Mean (±SD) use of over-ground robotic-assisted gait training device in week 1 and week 10.

		Week 1	Week 10
Outcomes	Days/week	6.1 ± 0.9	5.3 ± 0.8
	Average steps/day	887 ± 520	945 ± 542
	Average time/day (min)	50 ± 20	72 ± 41
	RPE	12.8 ± 2.2	10.4 ± 3.2
O-RAGT settings	Assistance	72 ± 3	47 ± 9
	Threshold	17 ± 8	38 ± 12

O-RAGT: Over-ground Robotic-Assisted Gait Training; RPE: Ratings of Perceived Exertion.

There were no statistical differences in any of the clinical functional outcomes at baseline (all *P* > 0.05). A Condition by Time interaction was observed for the Six Minute Walk Test *(P* < 0.001; $\eta_{p}^{2}$ = 0.27, Table 3), indicating that the change in mobility endurance from baseline to 10 weeks was statistically greater in the over-ground robotic-assisted gait training group. This change was maintained at 22 weeks where it was still found to be statistically significant. Similar statistically significant differences were also observed for the Functional Ambulation Categories, Dynamic Gait Index, Berg Balance and for various accelerometry outcomes between baseline and the 10-week assessment (all *P* < 0.05; Tables 3 and 4). Improvements in the Functional Ambulation Categories, Dynamic Gait Index and Berg Balance scores were observed for the over-ground robotic-assisted gait training group compared to the control group ( $\eta_{p}^{2}$ = 0.18 to 0.28). For the accelerometry outcomes, the over-ground robotic-assisted gait training group demonstrated a significant decrease in time spent seated/supine, but increases in time spent stepping, number of steps and number of sit-to-stand transitions (all *P* < 0.05; Table 4). There were no further changes in any of the measures between the 10- and 22-week assessments for both Conditions (*P* > 0.05).

**Table 3.** Mean (±SD) scores from functional ambulation categories, dynamic gait index, berg balance and timed-up-and-go for over-ground robotic-assisted gait training and control from baseline, 10 weeks and 22 weeks after baseline.

		Assessment			Condition × time interaction	
		Baseline	10 weeks	22 weeks	*P*	$\eta_{p}^{2}$
6MWT (m)	O-RAGT	135 ± 81	158 ± 93	161 ± 91	0.000*	0.27
	CON	122 ± 92	119 ± 84	115 ± 83		
6MWT (RPE)	O-RAGT	12.8 ± 3.2	12.2 ± 3.0	12.6 ± 2.5	0.658	0.02
	CON	11.7 ± 3.1	11.9 ± 2.7	11.4 ± 2.4		
FAC	O-RAGT	3.4 ± 1.0	3.8 ± 0.9	3.8 ± 0.9	0.010*	0.18
	CON	3.4 ± 1.1	3.3 ± 1.1	3.2 ± 1.1		
DGI	O-RAGT	10.7 ± 3.3	13.1 ± 4.7	14.0 ± 3.6	0.003*	0.19
	CON	12.6 ± 5.7	12.7 ± 5.6	13.0 ± 4.5		
BBS	O-RAGT	40.9 ± 9.6	45.5 ± 9.0	45.6 ± 9.1	**0.000** *	0.28
	CON	43.3 ± 7.3	42.7 ± 7.4	43.6 ± 8.1		
TUG (s)	O-RAGT	36.2 ± 20.2	34.0 ± 19.1	33.2 ± 21.8	0.876	0.01
	CON	36.0 ± 21.6	32.9 ± 20.1	31.5 ± 20.5		

6MWT: Six Minute Walk Test; BBS: Berg Balance Scale; CON: Control; DGI: Dynamic Gait Index; FAC: Functional Ambulation Categories; O-RAGT: Over-ground Robotic-Assisted Gait Training; RPE: Ratings of Perceived Exertion; TUG: Time-Up-And-Go Test.

* Significant condition x time interaction (*P* less or equal than 0.05)

**Table 4.** Mean (±SD) accelerometry data for over-ground robotic-assisted gait training and control at baseline, 10 weeks and 22 weeks after baseline.

		Assessment			Condition × time interaction	
		Baseline	10 weeks	22 weeks	*P*	$\eta_{p}^{2}$
Time spent seated/supine (%)	O-RAGT	86.3 ± 10.6	83.4 ± 11.2	85.2 ± 9.6	0.050*	0.12
	CON	81.8 ± 8.3	83.1 ± 8.3	82.6 ± 8.1		
Time spent standing (%)	O-RAGT	10.5 ± 7.9	11.5 ± 8.3	10.3 ± 6.9	0.232	0.06
	CON	14.5 ± 5.7	13.1 ± 6.1	15.1 ± 7.9		
Time spent stepping (%)	O-RAGT	3.2 ± 3.0	5.2 ± 3.3	4.5 ± 3.1	0.009*	0.22
	CON	4.4 ± 2.6	3.8 ± 2.5	4.0 ± 2.7		
Number of steps (*n*)	O-RAGT	2754 ± 2809	4484 ± 3192	4105 ± 3350	**0.021** *	0.15
	CON	3412 ± 2456	3046 ± 2322	3274 ± 2960		
Number sit-to-stand transitions (*n*)	O-RAGT	34 ± 11	45 ± 19	43 ± 16	**0.011** *	0.17
	CON	45 ± 15	43 ± 15	43 ± 15		

CON: Control; O-RAGT: Over-ground Robotic-Assisted Gait Training.

* Significant condition x time interaction (*P* less or equal than 0.05)

Of the 14 over-ground robotic-assisted gait training participants who used a walking aid at baseline, two did not use a walking aid at the 10-week assessment during the Six Minute Walk Test and Timed-Up-and-Go test. There were no changes in the use of walking aids for control participants.

## Discussion

This study demonstrated improvements in clinical functional outcomes in chronic stroke survivors following a combination of daily, home-based, rehabilitation program using over-ground robotic-assisted gait training, in the form of a wearable robotic knee orthosis and usual care physiotherapy. Improvements were observed in walking ability, as determined by the Six Minute Walk Test and Dynamic Gait Index, and balance for those in the over-ground robotic-assisted gait training group. The observed reduction in sedentary behavior is an important positive implication when considering over-ground robotic-assisted gait training for “at home” rehabilitation therapy for stroke survivors.

The minimum clinical difference for a change in the Six Minute Walk Test is ⩾13%.^16^ The current study demonstrated a 15% improvement in Six Minute Walk Test between the baseline and 10-week assessments for participants undertaking over-ground robotic-assisted gait training. This improvement was maintained at the 22-week assessment. Improvements in walking ability is one of the most frequently demanded goals of rehabilitation and has been directly related to improvements in post-stroke quality of life.^24^ Although participants in the control group engaged in weekly, “usual care” physiotherapy sessions, a 3% decline in Six Minute Walk Test distance was observed for this group. Despite the statistical improvement in the Six Minute Walk Test distance for those in the over-ground robotic-assisted gait training group, there were no changes in participants’ RPE (Table 3). This implies that participants found the Six Minute Walk Test to be relatively easier at a higher intensity following the over-ground robotic-assisted gait training program compared to baseline.

In this study, participants wore the over-ground robotic-assisted gait training device for 56 days per week and for >50 minutes per day, for 10 weeks, demonstrating excellent program adherence within a home-based environment. At the end of the 10-week over-ground robotic-assisted gait training program, participants were wearing the robotic device for ~22 minutes more per day when compared to the start. As the training principle of progressive overload was implemented in the over-ground robotic-assisted gait training program, the small increase in step count at the end of the program compared to the start is likely due to the increase in exercise intensity, as determined by large reductions in the “Assistance” setting and pronounced increases in the “Threshold” setting over the course of the 10-week program.

Our study population (less able stroke patients) and exercise dosage (comparatively elevated) differs to that reported elsewhere when similar over-ground robotic-assisted gait training devices have been implemented.^4–7^ These previous studies reported no statistical improvements in Six Minute Walk Test following a three times per week, 4- to 6-week over-ground robotic-assisted gait training program in a clinical setting. This suggests that the amount of over-ground robotic-assisted gait training may have been inadequate to elicit statistical improvements in clinical functional outcomes in comparison to a non- over-ground robotic-assisted gait training group.^7^ The “at home” nature of our study and device accessibility may have enabled participants to undertake a higher volume of walking, as the participants could wear the over-ground robotic-assisted gait training device at any time or day during the program period. Participants had the potential to train at a faster cadence for more hours per week and utilize the over-ground robotic-assisted gait training device for a longer period of time (10 weeks) than that previously observed in the literature.

There was, on average, a 10% improvement (4.6 points) in Berg Balance Scale following the over-ground robotic-assisted gait training. The changes observed in our study were approximately twice as large as that observed in past research with similar robotic gait trainers.^7^ This difference may again be attributed to the greater accessibility and exercise dosage afforded by home-based over-ground robotic-assisted gait training program.

There were no differences in Timed-Up-and-Go performance between over-ground robotic-assisted gait training and control groups with improvements reported between baseline and 10 weeks, regardless of the condition. There was a wide variability in Timed-Up-and-Go performance (Table 3) and slower Timed-Up-and-Go completion times when compared to other research with a chronic stroke population (approx. 22 s).^25^ It is plausible that this measure was not sensitive enough to detect a meaningful change and the variation in our stroke population’s Functional Ambulation Categories may be one reason for this finding.

Participants’ habitual activity patterns, determined by accelerometry, positively changed for over-ground robotic-assisted gait training. In our study, there were significant reductions in the time spent seated and increases in time spent stepping for over-ground robotic-assisted gait training participants. Participants undertook an additional ~1700 steps per day at the time of the 10-week assessment compared to baseline (~39% improvement), which was maintained at the 22-week assessment. For the control group, 400 fewer steps on average were recorded at 10 weeks compared to baseline (−12%).

An important characteristic of successful behavior change is that individuals continue to engage in lifestyle modifications once the stimulus has been removed. A meta-analysis for stroke patients revealed that end-of-intervention benefits gained from regular physical fitness training do not persist after an intervention has ceased.^26^ The current study demonstrated, however, that the improvements reported in Six Minute Walk Test at 10 weeks were maintained at the 22-week assessment. Future research should assess the impact of over-ground robotic-assisted gait training on clinical functional outcomes over a longer follow-up period (i.e., 12-month follow-up).

It is important to highlight the limitations, strengths and practical implications of this research. Firstly, although pre-trial calculations suggested adequate power, the present study did have a small sample size which did experience some participant attrition (*n* = 3) between the 10- and 22-week assessment for the control group. Larger sample sizes are needed in future research to draw firm conclusions on results found.^27^ Secondly, participants were recruited from a private neuro-physiotherapy practice which could be a determining factor to whether a home-based program is successful. The selected population were likely to be highly motivated to engage in rehabilitation due to the costs associated with engaging in physiotherapy with a private provider.

Despite statistical improvements between baseline and 10 weeks, some outcome measures did not reach the minimal clinically important difference. For example, a change of 4.6 points was seen in the Berg Balance Scale in the over-ground robotic-assisted gait training group between baseline and 10 weeks. Stevenson^20^ however reported that a minimal clinically important difference of 7 points is necessary when implementing the Berg Balance Scale with stroke patients. As the 6MWT was the study’s primary outcome measure, findings with secondary outcomes such as the Berg Balance Scale must be interpreted with caution due to being exploratory in nature. Regardless of this, when making evidence-based clinical decisions on the effectiveness of implementing over-ground robotic-assisted gait training programs, both statistical findings and minimal clinically important difference should be considered when determining the efficacy of a rehabilitation program. Lastly, the study did not examine whether there were any mechanistic effects of the over-ground robotic-assisted gait training program on kinematic gait patterns.

A major strength of our study was the successful implementation of a home-based over-ground robotic-assisted gait training program. Over-ground robotic-assisted gait training may enable practitioners to increase the intensity and total duration of physical activity in either a clinical or home-based setting significantly benefitting individuals living with stroke. Reductions in sedentary time and an increase in physical activity could help prevent secondary complications associated with cardiovascular disease and future cardio- or cerebrovascular events (i.e., reducing strokes) if such programs are implemented over the longer-term.

This technology may be practical in terms of application in medical centers and community settings, however the cost is high and at present unlikely to meet the threshold for funding within the NHS without further evidence. The short- and longer-term implications of over-ground robotic-assisted gait training must be examined with larger and more representative populations to further establish optimal rehabilitation recommendations for stroke survivors. For example, our study sample was recruited from a single neuro-physiotherapy practice whereby participants largely fund their own treatment. Future research should also focus on implementing over-ground robotic assisted gait training interventions with acute stroke patients (⩽ six months), and consider the use of such devices for individuals who do not receive ongoing rehabilitation.

In conclusion, the present study has demonstrated that participation in a 10-week, home-based, over-ground robotic-assisted gait training program, in combination with weekly, usual care physiotherapy, can elicit improvements in clinical functional outcomes in patients with stroke. Importantly, the changes reported in clinical functional outcomes were maintained at a 22-week assessment. Individuals randomized to the over-ground robotic-assisted gait training program also demonstrated increases in physical activity and reductions in sedentary time, which could have the potential to improve quality of life. These findings collectively support the recommendation for implementing “at home” over-ground robotic-assisted gait training as a part of the stroke treatment pathway.

**Clinical messages**

- Home-based over-ground robotic-assisted gait training program combined with standard physiotherapy elicits significant improvements in functional outcomes and physical activity in chronic stroke patients compared to physiotherapy alone.
- Acute changes in clinical functional outcomes are maintained for at least 12 weeks after completing a 10-week, home-based over-ground robotic-assisted gait training program.

## Supplemental Material

sj-pdf-1-cre-10.1177_0269215520984133 – Supplemental material for Effect of combined home-based, overground robotic-assisted gait training and usual physiotherapy on clinical functional outcomes in people with chronic stroke: A randomized controlled trial Click here for additional data file. Supplemental material, sj-pdf-1-cre-10.1177_0269215520984133 for Effect of combined home-based, overground robotic-assisted gait training and usual physiotherapy on clinical functional outcomes in people with chronic stroke: A randomized controlled trial by Amy Wright, Keeron Stone, Louis Martinelli, Simon Fryer, Grace Smith, Danielle Lambrick, Lee Stoner, Simon Jobson and James Faulkner in Clinical Rehabilitation
