# Supplementary material for: Effect of combined home-based, overground robotic-assisted gait training and usual physiotherapy on clinical functional outcomes in people with chronic stroke: A randomized controlled trial
Source: Clin Rehabil. 2020 Dec 27;35(6):882–93. doi: 10.1177/0269215520984133 (PMC8191155; doi:10.1177/0269215520984133)
Supplement: sj-pdf-1-cre-10.1177_0269215520984133 – Supplemental material for Effect of combined home-based, overground robotic-assisted gait training and usual physiotherapy on clinical functional outcomes in people with chronic stroke: A randomized controlled trial [file sj-pdf-1-cre-10.1177_0269215520984133.pdf]

Supplementary Information

Alter G Bionic leg orthosis

The range of motion and amount of assistance the leg gives the user is customizable through a range or device settings. The settings are summarized below:

Assistance refers to the amount of support the device provides to the user to help with extension of the lower extremity. This is programed as a percentage of the individual's single-limb bodyweight, whereby a higher value demonstrates a greater contribution from the robotic device.

Threshold refers to the percentage of single limb body weight that must be applied to the footplate before assistance will be provided by the device. This is programed as a percentage of the individual's single-limb bodyweight, whereby a lower value demonstrates a greater contribution from the robotic device (i.e., it is more sensitive to small weight shifts.)

Resistance refers to the support provided during descent (flexion moment) that a user will experience during such tasks as sitting down or descending stairs. This setting remained the same throughout the 10-week program, as per manufacturers' guidelines.

Knee extension refers to the degrees from full extension to which the device will provide assistance and is comfortable for the patient. This setting remained the same throughout the 10-week program, as per manufacturers' guidelines.
